# Expression Dynamics of CA IX Epitope in Cancer Cells under Intermittent Hypoxia Correlates with Extracellular pH Drop and Cell Killing by Ureido-Sulfonamide CA IX Inhibitors

**DOI:** 10.3390/ijms24054595

**Published:** 2023-02-27

**Authors:** Md. Abu Sufian, Sabina Zamanova, Ahmed M. Shabana, Brianna Kemp, Utpal K. Mondal, Claudiu T. Supuran, Marc A. Ilies

**Affiliations:** 1 Department of Pharmaceutical Sciences and Moulder Center for Drug Discovery Research, School of Pharmacy, Temple University, 3307 North Broad Street, Philadelphia, PA 19140, USA; 2 Department of Pharmaceutics and Industrial Pharmacy, Faculty of Pharmacy, Cairo University, Cairo 11562, Egypt; 3 NEUROFARBA Department, Pharmaceutical Sciences Section, Universita degli Studi di Firenze, Polo Scientifico, Via Ugo Schiff No. 6, 50019 Sesto Fiorentino, Florence, Italy

**Keywords:** cancer, tumor, hypoxia, carbonic anhydrase IX, intermittent hypoxia, pH, inhibitor, growth, viability

## Abstract

Carbonic anhydrase IX (CA IX) is a membrane-bound CA isozyme over-expressed in many hypoxic tumor cells, where it ensures pH homeostasis and has been implicated in tumor survival, metastasis and resistance to chemotherapy and radiotherapy. Given the functional importance of CA IX in tumor biochemistry, we investigated the expression dynamics of CA IX in normoxia, hypoxia and intermittent hypoxia, which are typical conditions experienced by tumor cells in aggressive carcinomas. We correlated the CA IX epitope expression dynamics with extracellular pH acidification and with viability of CA IX-expressing cancer cells upon treatment with CA IX inhibitors (CAIs) in colon HT-29, breast MDA-MB-231 and ovarian SKOV-3 tumor cell models. We observed that the CA IX epitope expressed under hypoxia by these cancer cells is retained in a significant amount upon reoxygenation, probably to preserve their proliferation ability. The extracellular pH drop correlated well with the level of CA IX expression, with the intermittent hypoxic cells showing a similar pH drop to fully hypoxic ones. All cancer cells showed higher sensitivity to CA IX inhibitors (CAIs) under hypoxia as compared to normoxia. The tumor cell sensitivity to CAIs under hypoxia and intermittent hypoxia were similar and higher than in normoxia and appeared to be correlated with the lipophilicity of the CAI.

## 1. Introduction

**Figure 1:**
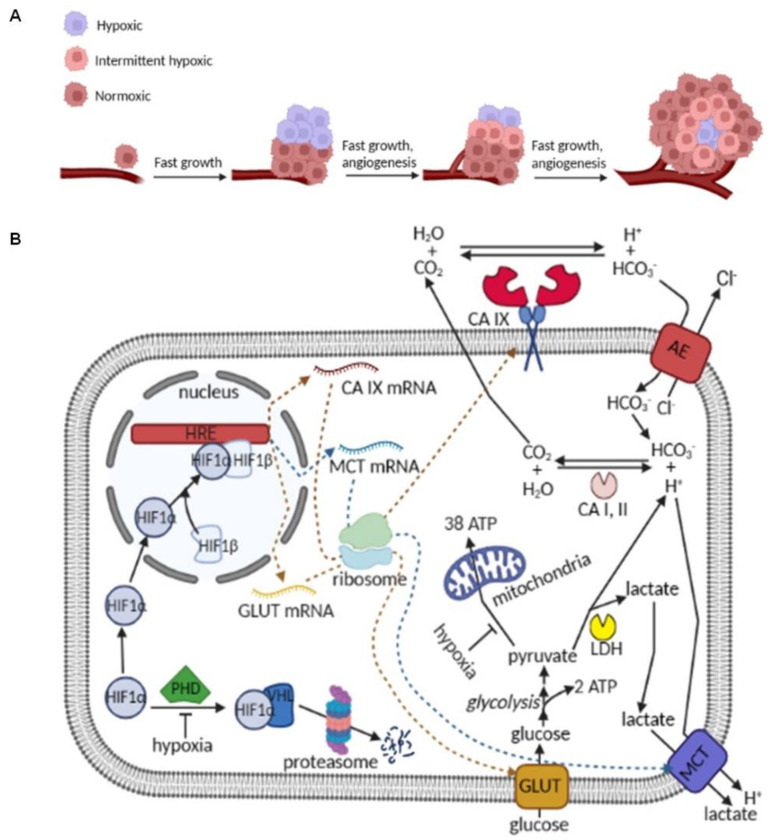
Cartoon depicting the tumor dynamics and microenvironment and highlighting normoxic, intermittent hypoxic and hypoxic area (**A**) and the genotypic and phenotypic changes associated with pH regulation in hypoxic and intermittent hypoxic tumor cells (**B**). Hypoxia stabilizes cytosolic HIF-1α, which translocates to the nucleus and forms a heterodimer with constitutively stable HIF-1β. Together, they upregulate the expression of GLUTs, HK, PDK-1, LDH, MCTs and CA IX, among many other genes required for cell adaptation to hypoxia. Glucose transporters (GLUT-1 and GLUT-3); glycolytic enzymes (HK, PDK-1 and LDH) and monocarboxylate transporters (MCT-1 and MCT-4) are significantly upregulated, as cells rely heavily on the anaerobic glycolytic pathway (upregulated 10-15X) for their ATP needs. In tandem with cytosolic carbonic anhydrase isozymes CA I and CA II, CA IX plays a major role in maintaining the intracellular pH homeostasis in tumor cells, acidifying the extracellular milieu.

Genetic and epigenetic mutations can transform the normal cells of the body into malignant ones that lack contact inhibition and start to grow in an aggressive, uncontrolled manner, forming tumors [1,2]. Many solid tumors grow following a Gompertzian growth pattern [3], consisting of an initial exponential growth, with low mutation rate and high cell homogeneity, followed by a slower growth phase, with low growth, high mutation rate and a high cell heterogeneity. Among the many factors that define this growth, a key one is the access of cells to nutrients and especially oxygen [4]. Initially, as tumor cells are located close to the vasculature, they receive glucose and oxygen from the blood supply, similar to neighboring normal cells. They multiply fast and display a high homogeneity. With continuing growth, some cells within the tumor are located farther from the vasculature and become hypoxic (Figure 1A) [4,5,6,7]. They have a variable exposure to oxygen, depending on their distance from the nearest blood vessel, as well as the partial pressure of oxygen PO_2_ in the immediate microvasculature feeding them [8]. These tumor cells oftentimes experience perfusion-limited hypoxia, which is acute, transient or intermittent (intermittent hypoxia) and caused by fluctuating blood flow within the leaky and immature blood vessels nourishing the heavy demand and very dynamic tumor microenvironment [7]. Under intermittent hypoxia, the growth of these tumor cells depends largely on the fluctuating PO_2_ they experience and on their ability to adapt to hypoxia by changing their biochemical metabolism [6,7,9] (Figure 1). Consequently, the rate of growth of the tumor decreases and mutation rate increases, favoring the clones resistant to the hypoxic environment. When the distance from a tumor cell to the nearest blood vessel is bigger than 150 µm, the local partial pressure of oxygen PO_2_ drops to zero. Cells located beyond this limit are extremely hypoxic and can undergo apoptosis and necrosis, thus generating the necrotic cores observed in solid tumors (Figure 1A) [9,10].

When exposed to hypoxia, many solid tumor cells upregulate hypoxia inducible transcription factor-1 (HIF-1)-mediated pro-survival and pro-metastatic pathways [11,12]. HIF-1 is a heterodimer, composed of an oxygen-sensitive α subunit, located in the cytosol, and a constitutively stable β subunit, located in the nucleus. In normal cells, as well as in tumor cells under normal oxygen exposure (normoxia), cytoplasmic HIF-1α is continuously hydroxylated by prolyl hydroxylase domain (PHD) proteins using molecular oxygen, and hydroxylated HIF-1α is identified by the tumor suppressor protein von Hippel-Lindau (VHL) factor. The HIF-1α-VHL complex subsequently undergoes proteasomal degradation by ubiquitination [13,14]. Under hypoxia, the hydroxylation of HIF-1α is reduced, and its concentration in cytoplasm increases, favoring the translocation to the nucleus, where it forms a heterodimer with the HIF-1β subunit [15,16]. This HIF complex binds upstream of genes bearing a hypoxia-responsive element (HRE) and triggers the expression of more than 500 proteins involved in pro-survival and pro-metastasis pathways, including glycolysis, angiogenesis, erythropoiesis, pH regulation, etc. [7,11,12,17,18,19,20,21].

Thus, hypoxic tumor cells undergo a metabolic switch, upregulating glycolysis 10–15 times to compensate for cellular ATP needs, as oxidative phosphorylation in the mitochondria is significantly reduced. In parallel, HIF-induced upregulation of glucose transporters (GLUTs), particularly GLUT-1 and GLUT-3, massively increases glucose import to meet the elevated cellular demand for glucose [22]. Glycolytic enzymes, e.g., hexokinase (HK), pyruvate dehydrogenase kinase 1 (PDK-1) and lactate dehydrogenase (LDH), are also overexpressed via the HIF-1 pathway, facilitating the quick conversion of glucose to pyruvate with the production of ATP. Pyruvate is subsequently converted to lactate to regenerate NAD^+^ and thus allow glycolysis to continue at a fast pace. Large amounts of lactate and protons constitute the two byproducts of glycolysis that are expelled out of tumor cells via monocarboxylate transporters (MCT), particularly MCT-1 and MCT-4, and by the action of several carbonic anhydrase (CA) isozymes, with a major role played by a membrane-bound isozyme carbonic anhydrase IX (CA IX) [23,24]. Both MCTs and CA IX are overexpressed as a HIF-mediated adaptive response to tumor hypoxia [12,25,26,27,28,29].

CA IX is a tumor-associated dimeric membrane-bound CA isozyme, which readily equilibrates the extracellular CO_2_/HCO_3_^−^ pools, and, in tandem with intracellular CA isozymes, such as CA I and CA II, maintains the cytoplasmic pH homeostasis in cancer cells. Carbonic anhydrases (CAs) are zinc metalloproteins that catalyze the reversible hydration of CO_2_ to HCO_3_^−^ and H^+^ (Figure 1) [26,30,31]. This physiologic reaction is essential for maintaining pH homeostasis in different subcellular compartments, as well as in the extracellular milieu, in respiration, electrolyte secretion in different tissues and organs, in anerobic metabolism and in tumorigenesis [6,25,26,30]. In hypoxic tumors, the glycolytic protons are combined with cytoplasmic HCO_3_^−^ and transformed into CO_2_ and H_2_O through the actions of cytoplasmic CA I and CA II (both fast isozymes, similar to CA IX (k_cat_/K_m_ for CA I = 5 × 10^7^ M^−1^ s^−1^, k_cat_/K_m_ for CA II = 1.5 × 10^8^ M^−1^ s^−1^ and k_cat_/K_m_ for CA IX = 5.5 × 10^7^ M^−1^ s^−1^) [26,30,31] (Figure 1B). The CO_2_ produced flows freely through the plasma membrane, and in the extracellular milieu, it is rehydrated via CA IX to HCO_3_^−^ and H^+^. The HCO_3_^−^ ion is imported in the tumor cells in exchange of chloride ion through anion exchange transporters (AEs), closing the transport cycle, whereas the protons acidify the external milieu, with detrimental repercussions to normal cells surrounding the tumor [25,30,32,33,34,35]. Tumor acidification by CA IX is a major cause of resistance to chemotherapy and radiotherapy [6,25,34,36,37,38,39]. CA IX expression in normal tissues is limited to the gastrointestinal tract (epithelium of stomach, gallbladder and pancreatic ducts and some cells of the small intestine) [26,30,31].

Hypoxia and HIF-mediated upregulation of pro-survival pathways, including CA IX, is a common feature of many solid tumors [40,41,42], including colorectal, breast and ovarian carcinomas [7,12,34,43,44,45,46,47]. In a previous study, we observed that, when colorectal HT-29, breast MDA-MB-231 and ovarian SKOV-3 cancer cells are exposed to hypoxia in vitro, the cells elevate the level of CA IX expression as compared to normoxia [48]. The inhibition of CA IX was shown by us and by others to be an efficient strategy to shrink and kill hypoxic tumors [25,26,48,49,50,51,52,53,54,55,56,57,58,59,60,61], as well as targeting applications for imaging and drug delivery nanosystems [62,63,64,65]. Particularly efficient were aromatic and heterocyclic ureido-sulfonamides [57,66,67], with one representative, SLC-0111, promoted in Phase I/II clinical trials for the treatment of solid tumors [52,68,69]. As the CA IX epitope is instrumental for hypoxic tumor cells, we hypothesized that, once this epitope is expressed under hypoxia, it is retained by the tumor cells upon reoxygenation. Thus, here, we investigate the expression dynamics of the CA IX epitope in the same set of cancer cells in 2D cell cultures under intermittent hypoxia, along with persistent hypoxia and persistent normoxia as positive and negative controls, respectively.

## 2. Results and Discussion

### 2.1. Cells Growth Dynamics

**Figure 2:**
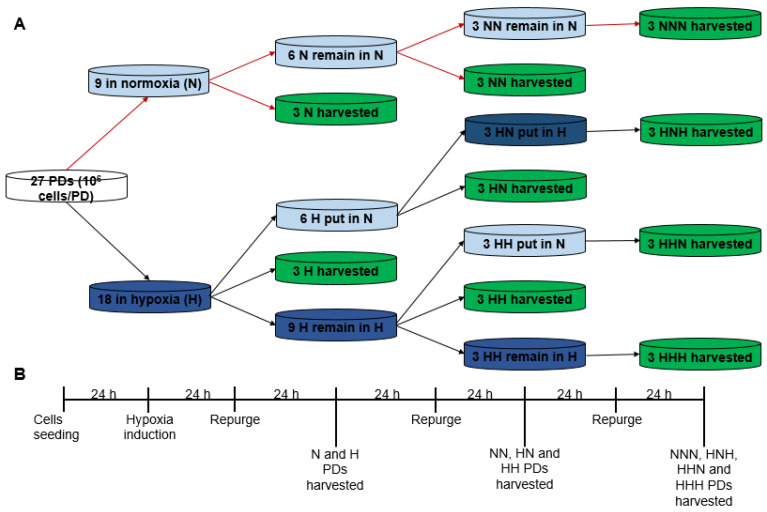
Schematic representation (**A**) and timeframe (**B**) of the cell growth dynamics experiment under normoxia (N, NN and NNN); hypoxia (H, HH and HHH) and intermittent hypoxia (HN, HNH and HHN) performed on colon HT-29, breast MDA-MB-231 and ovarian SKOV-3 cancer cells.

The growth dynamics of colon HT-29, breast MDA-MB-231 and ovarian SKOV-3 cancer cells were monitored under normoxia, hypoxia and intermittent hypoxia in a parallel experiment presented schematically in Figure 2. Cells from each line were cultured in 27 Petri dishes, beginning with 10^6^ cells in each dish, and were allowed to attach in normal conditions (37 °C, 5% CO_2_ in air). Subsequently, the Petri dishes were divided into three groups to allow cells growth under (a) normoxia (N: 2 days, NN: 4 days and NNN: 6 days), (b) hypoxia (H: 2 days, HH: 4 days, HHH: 6 days) and (c) intermittent hypoxia (HN: 2 days hypoxia followed by 2 days normoxia, HNH: 2 days hypoxia, followed by 2 days normoxia, followed by 2 days hypoxia, or HHN: 4 days hypoxia, followed by 2 days normoxia). At day 3, day 5 and day 7 of cell growth, HT-29, MDA-MB-231 and SKOV-3 cells from each group were harvested and counted (Figure 2).

**Figure 3:**
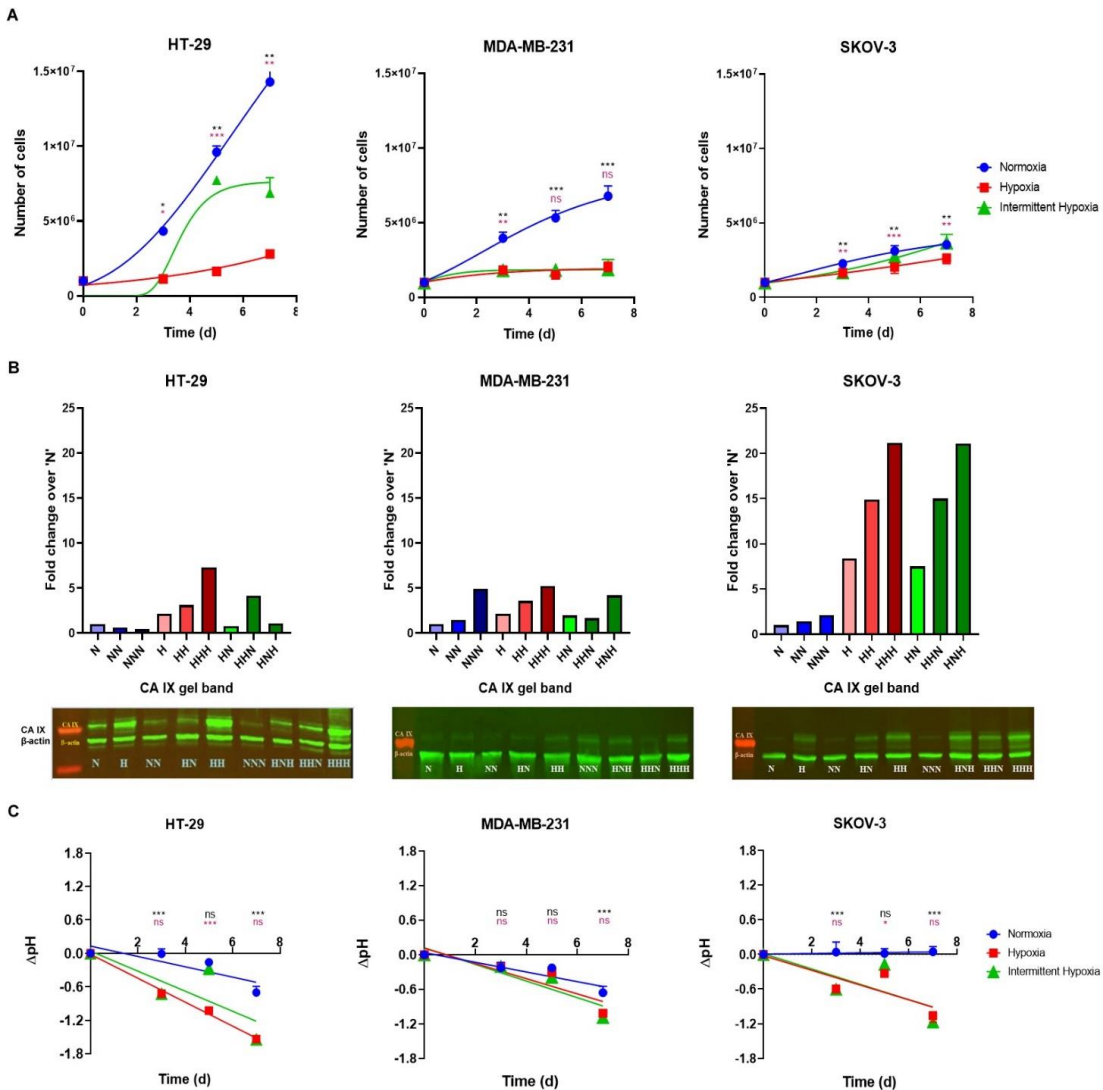
Cell growth dynamic (**A**), CA IX expression (**B**), with Western blot on the bottom and CA IX band signal integration on top) and extracellular pH drop in growth media (**C**) under normoxia (N, NN, NNN), hypoxia (H, HH, HHH) and intermittent hypoxia (HN, HNH and HHN) in HT-29, MDA-MB-231 and SKOV-3 cells. *p*-values were determined by one-way ANOVA. * *p* < 0.05, ** *p* < 0.01, *** *p* < 0.001 and ns = not significant. Black and pink stars indicate statistically significant difference in intermittent hypoxic and hypoxic groups respectively as compared to normoxic group.

The dynamics of cell growth under normoxia, hypoxia and intermittent hypoxia are presented in Figure 3. An analysis of Figure 3A revealed that, under standard normoxic conditions, the tumor cell growth was highly dependent on cell type, with the HT-29 colon cancer cells growing very aggressively (doubling time = 33.6 h), followed by MDA-MB-231 breast cancer cells (doubling time = 36.9 h) and SKOV-3 ovarian cancer cells (doubling time = 54.2 h). The number of HT-29 and MDA-MB-231 cells count significantly increased from N to NN to NNN, and within a week, starting from an initial 1 million cells, HT-29 and MDA-MB-231 cells multiplied to ~15 million and ~7 million cells, respectively. SKOV-3 cells, on the other hand, showed a slow growth pattern under normoxia, ending with about 3.5 million cells after 7 days (Figure 3A). On the other hand, hypoxic conditions inhibited cells proliferation across all cell lines. After one week growth under hypoxia, from an initial 1 million cells, HT-29, MDA-MB-231 and SKOV-3 cells multiplied to 2.8 million, 1.5 million and 1.7 million cells, respectively. One can observe that, even under hypoxia, the growth of HT-29 cells was significant and showed an increase throughout hypoxic exposure, from H to HH to HHH. Although MDA-MB-231 cells grew from H to HH to HHH, the growth was not very significant. Unlike HT-29 or MDA-MB-231 cells, the growth of SKOV-3 cells under hypoxic conditions was the least affected, these cells proliferating at a pace close to the growth rate observed under normoxic conditions (Figure 3A). Under intermittent hypoxia, the number of live HT-29 cells significantly increased from H to HN; however, when HN cells were returned to hypoxia, the observed cells growth was not significant. In the same growth conditions, MDA-MB-231 cells showed a limited (non-statistically significant) increase in the live cells count, whereas SKOV-3 cells demonstrated a statistically significant cells growth from H to HN to HNH (Figure 3A). When comparing the cell growth dynamics between the normoxic, hypoxic and intermittent hypoxic groups, one can observe that the number of live HT-29 cells count under intermittent hypoxic conditions was significantly higher than the number of hypoxic HT-29 cells, while significantly lower than the number of normoxic HT-29 cells at the three time points tested (day 3, day 5 and day 7). For MDA-MB-231 cells, the number of cells count was significantly lower in the intermittent hypoxic group as compared to the normoxic group, with no statistically significant difference observed between the hypoxic and intermittent hypoxic groups at the three time points tested. Opposite to MDA-MB-231 cells, no significant difference in growth was observed in between the normoxic and intermittent hypoxic groups of SKOV-3 cells at days 3, 5 and 7 of the experiment. However, the cell count in the intermittent hypoxic group was significantly higher than that of the hypoxic group at the same three time points tested (Figure 3A).

### 2.2. Level of CA IX Expression

We already established that HT-29, MDA-MB-231 and SKOV-3 cells express CA IX under hypoxic conditions in vitro [48]. The experiment described in Figure 2 allowed us to explore the expression dynamics of CA IX in these cell models under intermittent hypoxia in comparison with hypoxic and normoxic conditions. Thus, cells harvested after exposed to N, H, NN, HN, HH, NNN, HNH, HHN and HHH were lysed and screened for the level of CA IX expression via Western blotting, using β-actin as a house-keeping control. The gel image was analyzed using ImageJ software, and the area under the curve for each gel band was quantified (Figure 3B top, with actual gels on the bottom). An examination of Figure 3B revealed that a basal level of CA IX expression was maintained under normoxia (N, NN and NNN), which was relatively constant (from N to NN or NN to NNN). The level of CA IX expressed under normoxic conditions was cell-dependent, with the HT-29 colon cancer cell line expressing the highest epitope level. All three cell lines showed significant CA IX expression under hypoxic conditions, thus confirming our previous observations [48]. Noticeably, CA IX expression increased linearly with exposure time to hypoxia, from 2 days (H) to 4 days (HH) to 6 days (HHH) in all cell lines tested. HT-29 cells showed the highest level of CA IX expression under hypoxia (H, HH and HHH), followed by SKOV-3 and MDA-MB-231 cells, respectively. However, when comparing the expression of CA IX in hypoxia vs. the basal (normoxic) level, the biggest increase in CA IX expression was observed in SKOV-3 cells, followed by HT-29 and MDA-MB-231 cells. Thus, in SKOV-3 cells/HT-29/MDA-MB-231 CA IX expression increased 8-fold/4-fold/2-fold after 3 days of hypoxia (H), 15-fold/6-fold/3.5-fold after 5 days (HH) and 21-fold/7-fold/5-fold after 7 days (HHH), respectively, as compared to the corresponding normoxic cells (N) (Figure 3B). Importantly, we found than when hypoxic cells were reoxygenated (intermittent hypoxia, HN, HHN, HNH) the CA IX epitope was generally retained by cells in all three cell lines.

In SKOV-3 cell line, the CA IX was completely retained, while HT-29 and MDA-MB-231 cell lines had larger fluctuations of the epitope level. Interestingly, when SKOV-3 and MDA-MB-231 cells exposed to intermittent hypoxia re-experienced hypoxic conditions (HNH), the CA IX expression level increased further, reaching the expression level of the continuously hypoxic cells (HHH). The HT-29 cell line behaved a bit different, probably because this cell line already expressed the highest amount of CA IX epitope (Figure 3B).

### 2.3. Extracellular pH

In order to further support the switch in tumor biochemistry under hypoxic and intermittently hypoxic conditions, we measured the extracellular pH drop associated with the catalytic function of CA IX in the HT-29, MDA-MB-231 and SKOV-3 cells grown under normoxic, hypoxic and intermittent hypoxic conditions as mentioned above. Thus, at day 3, day 5 and day 7 of cells growth, cells growth media from each group of cells was collected, and pH was measured (prior to cell harvesting and counting, as described above) via a pH meter. The measured pH values were deducted from the pH of growth media without any cells that underwent the similar experimental conditions and the difference (ΔpH) was plotted as a function of time, for each cell line (Figure 3C). Upon examination of Figure 3C, one can observe that the pH drop under normoxia was correlated well with the growing rate of cells (compare Figure 3A with 3C). HT-29 and MDA-MB-231 cells showed a pH drop in extracellular growth media under normoxia from day 3 (N), to day 5 (NN), to day 7 (NNN). SKOV-3 cells, which grow at a slower pace on the other hand, did not cause any pH drop in the extracellular growth media under normoxia. In contrast, all three tumor cell lines showed a significant drop in extracellular pH under hypoxia (Figure 3C). The ΔpH increased linearly with the exposure time to hypoxia, from day 3 (H), to day 5 (HH), to day 7 (HHH) and correlates very well with the expression of CA IX epitope, further confirming the role of CA IX in tumor pH regulation (Figure 1B) and the findings of other groups [25,30,32,33,34,35]. Extremely interesting was the ΔpH displayed by the three tumor cell lines under intermittent hypoxia, which was almost mirroring the hypoxia data. These values correlated very well with the expression of CA IX isozyme (compare Figure 3C with Figure 3B), further demonstrating the key role played by this CA isozyme in tumor metabolism under hypoxia and intermittent hypoxia.

Taken altogether, data from Figure 3 support the fast switch in tumor biochemistry (Figure 1B) under oxygen depletion (hypoxia) and the importance of CA IX epitope in this context. It proves that once the metabolic switch has occurred, the cells tend to conserve the hypoxic phenotype and the CA IX isozyme even after reoxygenation (intermittent hypoxia or even normoxia), as a key advantage and adaptation to any future hypoxic events. This ensures the (slower but) continuous growth of tumor cells in conditions of low or fluctuating PO_2_ and supports the growth dynamic recorded for these three carcinomas. The retention of the hypoxic phenotype ensures the acidification of extracellular milieu, slowly killing the normal cells surrounding the tumor and making room for tumor expansion in the neighboring tissues [25,30,32,33,34,35], as mentioned above. Tumor acidification by CA IX is also immunosuppressive and constitutes a major cause of resistance to chemotherapy and radiotherapy [6,25,34,36,37,38,39].

### 2.4. Cell Viability under Normoxia and Hypoxia

Since inhibition of CA IX was shown by us and by others to be an efficient strategy to shrink and kill hypoxic tumors [25,26,48,49,50,51,52,53,54,55,56,57,58,59,60,61], we investigated the impact of several potent CA IX inhibitors on the growth of the HT-29, MDA-MB-231 and SKOV-3 tumor cells under normoxic and hypoxic conditions.

**Figure 4:**
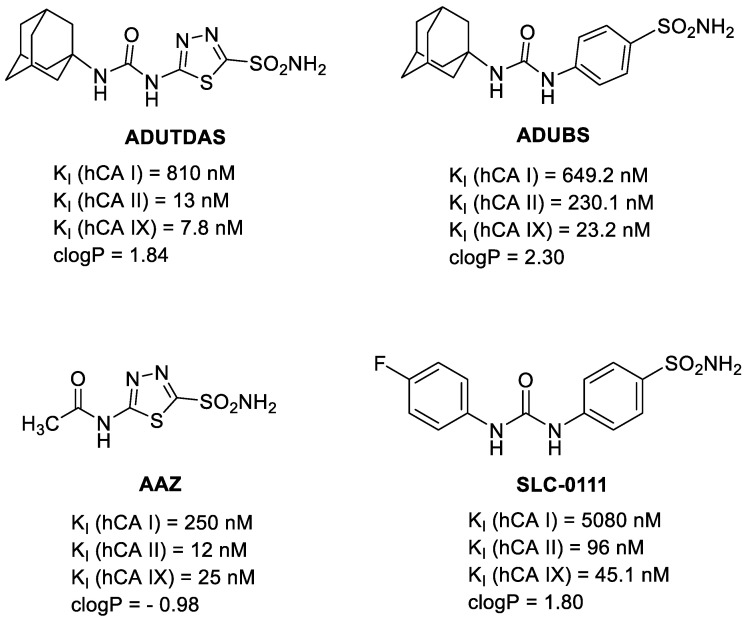
CA IX inhibitors used in this study [26,57,67,68,70], having different potencies against CA I, CA II and CA IX isozymes and different lipophilicities.

Thus, four CA IX inhibitors (Figure 4), two having a 1,3,4-thiadiazole sulfonamide warhead (ADUTDAS [67,70] and classical carbonic anhydrase inhibitor (CAI) acetazolamide AAZ) and two bearing a benzene sulfonamide warhead (ADUBDS [67] and SLC-0111 [57,68,69]) were tested against our HT-29, MDA-MB-231 and SKOV-3 cells comparatively, under normoxic and hypoxic conditions. Acetazolamide AAZ, which is used clinically, has a good inhibitory profile against both cytosolic CA I, CA II and against CA IX (Figure 4) [26] and was used in many studies as a reference CAI [25,26,48,49,50,51,52,53,54,55,56,57,58,59,60,61], while the SLC-0111 is the most advanced antitumoral CAI, being currently in Phase I/II clinical trials for the treatment of solid tumors [52,68]. Notably, SLC-0111 is less potent against CA IX than acetazolamide AAZ, but is more lipophilic, which makes it highly protein-bound, with benefic effects on its pharmacokinetic profile [68]. It belongs to the class of ureido-sulfonamides CAIs, introduced by us more than two decades ago [66]. The other two CAIs used in this study, adamantane derivatives ADUTDAS and ADUBS, belong to the same category and were proven to be potent inhibitors against CA IX (Figure 4), with similar or higher lipophilicity than SLC-0111 [67,70].

**Figure 5:**
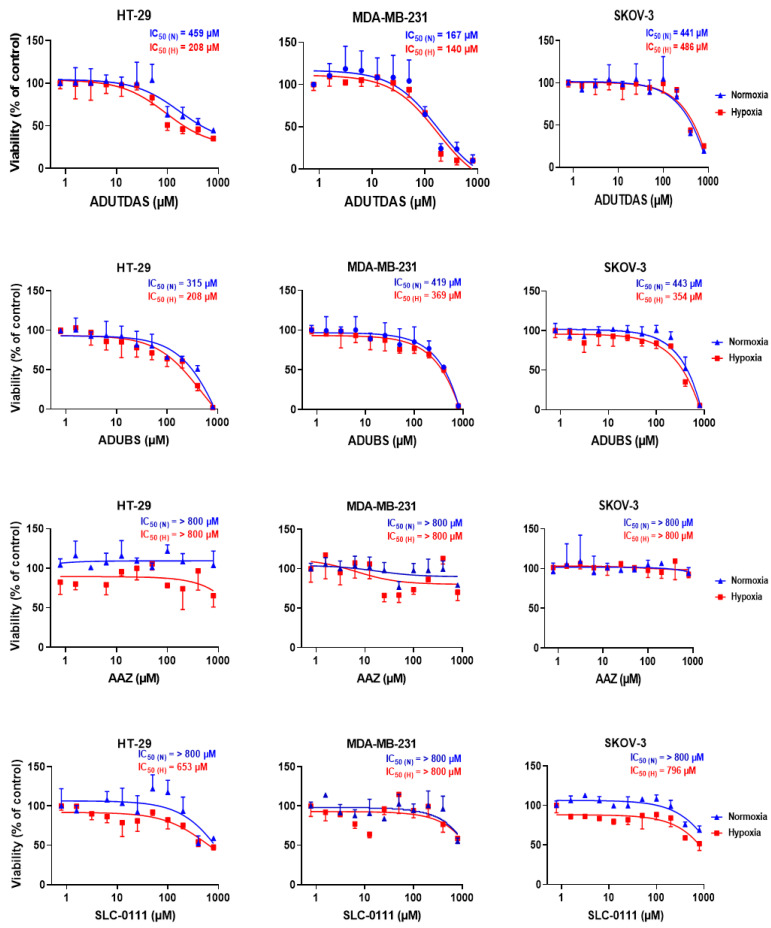
Viability of HT-29, MDA-MB-231 and SKOV-3 cells upon treatment with CAIs ADUTDAS, ADUBS, AAZ and SLC-0111 under normoxia (blue curves) and hypoxia (red curves). The IC_50_ values determined for each inhibitor were indicated.

HT-29, MDA-MB-231 and SKOV-3 tumor cells were cultured sub confluent in 96-well plates (10,000 cells/well), at 37 °C, 5% CO_2_ in air as mentioned above. After 24 h of growth at this condition, half of the plates for each cell line were moved to a hypoxia chamber and purged with hypoxia gas mix (1% O_2_, 5% CO_2_ and 94% N_2_), whereas the other half were kept in the same normoxic conditions for 48 h. CAIs stock solutions (2.5 mM) were prepared by diluting DMSO stock solutions of compounds with PBS. Cells were treated with CAI solutions at 11 different concentrations (800 µM, 400 µM, 200 µM, 100 µM, 50 µM, 25 µM, 12.5 µM, 6.25 µM, 3.125 µM, 1.56 µM and 0.78 µM) made from the CAI stock solutions via dilution with cell growth media in either normoxic or hypoxic conditions. After incubation with CAI solutions for 24 h, the viability of cells was measured using an MTT assay (Figure 5).

Data from Figure 5 revealed that CAIs affected the cancer cells viability in a concentration-dependent manner, under both normoxia and hypoxia, and that the sensitivity to CAIs was higher under hypoxia as compared to normoxia due to an over-expression of CA IX, as presented above (Figure 3B). Cells also showed sensitivity to CAIs under normoxia probably due to a basal level of CA IX present in these cell lines and to the inhibition of cytosolic isozymes CA I and CA II, also involved in pH homeostasis in tumor cells (Figure 1B). The sensitivity of cancer cells to CAIs was cell line dependent and inhibitor-dependent. Cell sensitivity was found to generally be HT-29 > SKOV-3 ≥ MDA-MB-231 (Figure 3B). Focusing on the potency of individual compounds against tumor cells, one can observe that the classical inhibitor acetazolamide AAZ displayed low cell killing efficacy both under normoxia and hypoxia against all cell lines, thus confirming previous finding from different research groups, including ours [25,26,48,49,50,51,52,53,54,55,56,57,58,59,60,61]. The clinically investigated CA IX inhibitor SLC-0111 showed a significantly better cell killing profile as compared to AAZ, especially under hypoxic conditions, with an IC_50_ of 653 µM, 796 µM and >800 µM for HT-29, SKOV-3 and MDA-MB231 cells. Interestingly, the adamantane CAI ADUTDAS, having a rather similar lipophilicity and CAI profile with SLC-0111, was able to reduce the viability of HT-29 colon cancer cells with an IC_50_ of 459 µM under normoxia, which decreased to 208 µM under hypoxia. However, CAI ADUTDAS was more efficient in killing MDA-MB-231 cells, showing an IC_50_ of 167 µM and 140 µM under normoxia and hypoxia, respectively. SKOV-3 cells had a different susceptibility for ADUTDAS, with an IC_50_ of 441 µM and 486 µM under normoxia/hypoxia. On the other hand, the IC_50_ of CAI ADUBS, which is less potent than ADUTDAS but more lipophilic, decreased from normoxia to hypoxia in all cell lines tested, showing the highest sensitivity against HT-29 cells (IC_50_ of 315 µM/208 µM under normoxia/hypoxia), followed by MDA-MB-231 (IC_50_ of 419 µM/369 µM under normoxia/hypoxia) and SKOV-3 cells (IC_50_ of 443 µM/354 µM under normoxia/hypoxia). Noticeably, for ADUBS, the drop in IC_50_ values was more pronounced against HT-29 and SKOV cells than in MDA-MB-231 cells, which could be due to a relative increase in CA IX from normoxia to hypoxia (8-fold in SKOV-3, 4-fold in HT-29 and only 2-fold for MDA-MB-231 cells). One can conclude that although the expression of CA IX epitope and the CA IX potency of the CAI are important for its efficiency in killing tumor cells, other factors such as lipophilicity of the CAI, specific protein expression (cell-dependent) can also play an important contribution in this direction. CAI ADUTDAS and ADUBS were more efficient than SLC-0111 in reducing the viability of all three tumor cell lines tested, under both normoxia and hypoxia (Figure 4).

### 2.5. Cell Viability under Intermittent Hypoxia

**Figure 6:**
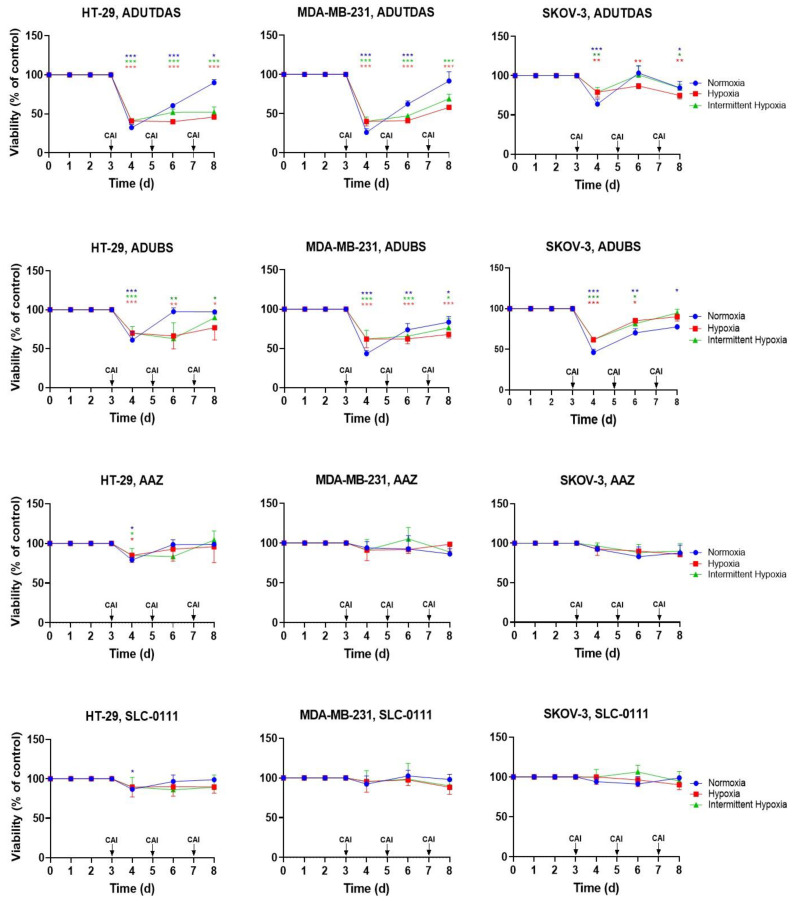
Viability of HT-29, MDA-MB-231 and SKOV-3 cells upon treatment with media containing 100 µM of ADUTDAS, ADUBS, AAZ or SLC-0111, at day 3, day 5 and day 7 of cell growth under normoxia, hypoxia and intermittent hypoxia. *p*-values were determined by one-way ANOVA. * *p* < 0.05, ** *p* < 0.01 and *** *p* < 0.001. Blue, red and green stars indicate statistically significant difference as compared to control within normoxic, hypoxic and intermittent hypoxic groups, respectively.

Having established the IC_50_ of the four CAIs against the cancer cells under normoxic and hypoxic conditions, we proceeded to examine the sensitivity of the same cancer cell lines against these CAIs dynamically, under normoxia, hypoxia and intermittent hypoxia. Thus, HT-29, MDA-MB-231 and SKOV-3 cells were cultured in 96-well plates (5000 cells/well) under normoxia, hypoxia and intermittent hypoxia as mentioned above (see Section 2.1 and Figure 2). At day 3, day 5 and day 7 of cell growth, cells in each group were treated with 100 µM CAI solutions in media and incubated in respective growth conditions. After incubation with CAI solutions for 24 h, the CAI solution in media was removed and the viability of cells was measured using an MTT assay (Figure 6). An analysis of data from Figure 6 revealed that treatment with CAIs (all 100 µM) was able to reduce the viability of the HT-29, MDA-MB-231 and SKOV-3 cells and that this reduction depended on the cell line, CAI used and specific conditions, as expected. Thus, the HT-29 cell line, which expressed the highest total amount of CA IX in both normoxia, hypoxia and intermittent hypoxia (Figure 3B), was the most susceptible to the treatment with all four CAI, which reduced its viability from day 3 to day 7 under hypoxia. The intermittent hypoxia, showed quite similar dynamics upon treatment with CAI, being well correlated with the expression of CA IX under these conditions (Figure 3B). The ability of CAIs to control the proliferation of cancer cells under normoxic conditions was gradually lost in time. We attribute this behavior to the lower level of CA IX expression under normoxic conditions.

The other two tumor cell lines behaved quite similarly with HT-29 under all conditions and CAI treatments (Figure 6), thus confirming previous observations [48,49,59]. Focusing on the potency of individual compounds, best results were obtained with CAI ADUTDAS, which was able to reduce the viability of HT-29/MDA-MB231/SKOV-3 cells to about 40%/35%/60% after 24 h incubation time with 100 µM CAI (day 4). It was followed by the CAI ADUBS, which was able to reduce the viability of HT-29/MDA-MB231/SKOV-3 cells to about 55%/50%/55%, by SLC-0111 and AAZ, with a reduction of viability of HT-29/MDA-MB231/SKOV-3 cells to about 80%/90%/90% in the same conditions. The two adamantane ureido-sulfonamides ADUTDAS and ADUBS were able to control (inhibit) the proliferation of the three tumor cell lines under all normoxia/hypoxia and intermittent hypoxia for another 4 days, being more efficient under hypoxia/intermittent hypoxia, in good correlation with the expression of CA IX epitope. The significant difference between the biological effect elicited by ADUTDAS and ADUBS pleads for the importance of moieties attached to the sulfonamide warhead in the classical “tail approach” for designing potent and efficient CAI [71], especially their lipophilicity [49,71] and warrant further investigations into this class of CAI for tumor cell treatment.

## 3. Materials and Methods

### 3.1. Materials

The following materials were used as received: Human colon adenocarcinoma cell line (HT-29), human breast cancer cell line (MDA-MB-231) and human ovarian cancer cell line (SKOV-3) were purchased from ATCC (Manassas, VA, USA). RPMI-1640, Dulbecco’s Modified Eagle’s Medium (DMEM), McCoy’s 5A media, trypsin-EDTA (0.05% trypsin, 0.53 nM EDTA) and Dulbecco’s Phosphate Buffer Saline (DPBS) were from Mediatech-Corning (Manassas, VA, USA). Fetal Bovine Serum (FBS) was from Rocky Mountain Biologicals (Missoula, MT, USA), RIPA buffer was from Amresco (Solon, OH, USA). 3-(4,5-Dimethylthiazol-2yl-)-2,5-diphenyltetrazolium bromide (MTT), and dimethyl sulfoxide (DMSO) was purchased from VWR International (West Chester, PA, USA). Acetazolamide was purchased form Sigma-Aldrich (St. Louis, MO, USA). The ureido-sulfonamide CAIs were synthesized as previously described [57,67].

### 3.2. Cell Culture

Cancer cells (HT-29, MDA-MB-231 and SKOV-3) were allowed to grow in RPMI-1640, DMEM and McCoy’s 5A media, respectively, supplemented with 10% FBS and 1% penicillin–streptomycin solutions. Cells were subcultured periodically in T-75/T-175 culture flasks, maintained in a humidified atmosphere containing 5% CO_2_, at 37 °C. Media was changed every other day.

### 3.3. Study of Cells Growth Dynamics under Normoxia, Hypoxia and Intermittent Hypoxia

Two-dimensional cell growth of HT-29, MDA-MB-231 and SKOV-3 was monitored according to Figure 2. The experiment was started with 27 × 10^6^ cells for each cell line. At day 0, cells were divided equally into 27 Petri dishes, with 10^6^ in each Petri dish, and allowed to attach under normoxic conditions. After 24 h (day 1), 9 Petri dishes were left under normoxic conditions, while 18 Petri dishes were moved into a hypoxic chamber, purged with a hypoxic gas mixture containing 1% O_2_, 5% CO_2_ and 94% N_2_ and kept under hypoxia for 48 h. To maintain hypoxic conditions, the chamber was repurged with hypoxia gas mixture after 24 h (day 2). After 48 h (day 3), 3 normoxic Petri dishes (N) and 3 hypoxic Petri dishes (H) were harvested, and live cells were counted using a hemocytometer. Media was changed from remaining 6 N and 15 H Petri dishes. Following media change, 6 N and 6 H (out of remaining 15 H) Petri dishes were returned to normoxia with labels ‘NN’ and ‘HN’ on, respectively. The remaining 9 H Petri dishes, denoted as ‘HH’, were returned to the hypoxic chamber, purged with hypoxia gas mixture and left under hypoxia for additional 48 h. After 24 h (day 4) the chamber was repurged with hypoxia gas mixture. After another 24 h (day 5), 3 NN, 3 HN and 3 HH Petri dishes were harvested, and live cells were counted using a hemocytometer. Media was changed from remaining 3 NN, 3 HN, 6 HH Petri dishes. Following medium change, 3 NN and 3 HH Petri dishes were returned to normoxia with labels ‘NNN’ and ‘HHN’ on, respectively. The remaining 3 HN and 3 HH Petri dishes, denoted as ‘HNH’ and HHH, were returned to the hypoxic chamber, purged with hypoxia gas mixture and left under hypoxia for additional 48 h. After 24 h (day 6) the chamber was repurged with hypoxia gas mixture. After another 24 h (day 7) 3 NNN, 3 HHN, 3 HNH and 3 HHH Petri dishes were harvested, and live cells were counted using a hemocytometer. Cells growth in normoxic, hypoxic and intermittent hypoxic group was presented as a function of time. Each data point was an average of three experiments, with one standard deviation from the average value. For intermittent hypoxic group, cells growth at day 7 was reported as an average of HNH (3 data points) and HHN (3 data points) groups. Statistical comparisons were performed by analysis of variance (ANOVA) using GraphPad Prism 8 (GraphPad Software, Boston, MA, USA). *p*-values * *p* < 0.05, ** *p* < 0.01, *** *p* < 0.001 and ns = not significant were considered.

### 3.4. Western Blotting

HT-29, MDA-MB-231 and SKOV-3 cells were cultured under normoxia, hypoxia or intermittent hypoxia as mentioned above. N, H, NN, HN, HH, NNN, HNH, HHN and HHH cells were harvested, pelleted, washed with PBS and then lysed with RIPA buffer containing protease inhibitors. Cell lysates were collected and stored at −20 °C until assessed. Total protein concentration was determined using bicinchoninic acid assay (BCA) method. The level of expression of CA IX under normoxia, hypoxia and intermittent hypoxia was determined by Western blotting as previously described [48]. Thus, appropriate volumes of collected cell lysates (10 µL containing 15 µg of total protein) were loaded onto 10% SDS-PAGE precast gels (Mini-PROTEAN TGX precast gels, Bio-Rad, Hercules, CA, USA), together with a Chameleon 700 pre-stained protein ladder (5 µL) (Li-Cor Biosciences, Lincoln, NE, USA), and protein separation was done at 150 V for 1 h. Gels were transblotted onto Odyssey nitrocellulose membranes (7.0 cm × 8.5 cm) (Li-Cor Biosciences, Lincoln, NE, USA) at 100 V for 30 min, and membranes were blocked using blocking buffer (Li-Cor Biosciences, Lincoln, NE, USA) for 1 h, followed by incubation with a specific primary mouse monoclonal antibody for CA IX (M 75 from Bioscience Slovakia, Bratislava, SK) or CA IX (H-11 from Santa Cruz Biotechnology (sc-365900), Dallas, TX, USA) at 4 °C overnight. After washing, membranes were incubated with anti-mouse IgG IRDye800CW secondary antibody (Li-Cor Biosciences, Lincoln, NE, USA) for 1 h at room temperature. Finally, membranes were visualized using an Odyssey image system (Li-Cor Biosciences, Lincoln, NE, USA). Beta actin was used as a control and was detected using a mouse monoclonal beta actin antibody (AC-15 GTX 26276, GeneTex, Irvine, CA, USA) binding, followed by detection with the same IgG IRDye800CW secondary antibody.

### 3.5. Measurement of Extracellular pH

To measure extracellular pH, HT-29, MDA-MB-231 and SKOV-3 cells were cultured in Petri dishes under normoxia, hypoxia and intermittent hypoxia, as mentioned above. At day 3, day 5 and day 7 of cell growth, cell growth media of N and H (day 3); NN, HN and HH (day 5); NNN, HNH, HHN, and HHH (day 7) designated PDs, respectively, were collected. Dead cells were pelleted out, and the supernatant was collected, and the pH was measured using an ORION STAR A211 pH meter (Thermo Scientific, Waltham, MA, USA). In parallel, media was collected from control Petri dishes (N, and H (day 3); NN, HN and HH (day 5); NNN, HNH, HHN and HHH (day 7)) containing only media, and pH was measured. Prior to pH measurement, the instrument was calibrated with three standard buffer solutions of known pH. At each time point, ΔpH was calculated by subtracting the pH values of growth media from the cell supernatant. Data were reported as average ΔpH ± SD, where each data point was an average of three experiments. For intermittent hypoxic group, ΔpH at day 7 was reported as an average of HNH (3 data points) and HHN (3 data points) groups. Statistical comparisons were performed by analysis of variance (ANOVA) using GraphPad Prism 8. *p*-values * *p* < 0.05, ** *p* < 0.01, *** *p* < 0.001 and ns = not significant were considered.

### 3.6. Cell Viability Assay

Cytotoxicity of ADUTDAS, ADUBS, acetazolamide (AAZ), and SLC-0111 was tested separately under normoxia and hypoxia against HT-29, MDA-MD-231 and SKOV-3 cell lines via an MTT assay as previously described [48]. Cells were plated in 96-well plates at a density of 10,000 cells/well and were allowed to attach and to grow in normal conditions (37 °C, 5% CO_2_ in air) for 24 h. After 24 h of growth in normal conditions, the hypoxia designated plates were placed in a hypoxic chamber, which was purged and filled with a hypoxic gas mixture (1% O_2_, 5% CO_2_ and 94% N_2_) to induce hypoxia. The closed chamber with hypoxic plates were kept in the incubator at 37 °C for 48 h. After hypoxia induction, both normoxic, and hypoxic plates were retrieved, media was aspirated, and cells were treated with different concentrations (800 µM, 400 µM, 200 µM, 100 µM, 50 µM, 25 µM, 12.5 µM, 6.25 µM, 3.125 µM, 1.56 µM or 0.78 µM) of CAIs solutions, made from CAIs stock solutions (2.5 mM) via dilution with cell growth media. CAIs stock solutions were prepared by diluting DMSO stock solutions of CAIs with PBS. Each experiment was carried out in quadruplicate. In each plate, eight wells with cells served as controls that received only media. Following treatment, normoxic plates were returned to normoxic conditions, whereas hypoxic plates were placed back to the hypoxic chamber and purged with the hypoxic gas mixture. The chamber was purged with hypoxic gas mixture every 24 h until the hypoxic plates were assessed for cell viability. After 24 h of treatment with CAI solutions, plates were retrieved, media was aspirated and cells were washed once with phosphate-buffered saline (PBS), which was subsequently removed. An MTT solution in media (120 μL), prepared from an MTT stock solution (5 mg/mL) in PBS via a 1:6 dilution with growth media, was added to each well, and the plates were incubated for 4 h at 37 °C. After 4 h, the MTT solution was removed, 150 μL of DMSO/well was added to solubilize the blue formazan crystals and then plates were incubated at 37 °C for 5 min. Then, the plates were analyzed spectrophotometrically using SpectraMax M2. Absorbance was measured at 570 nm, with a reference absorbance at 690 nm that was subtracted from the readings. Data was reported as the average of four experiments, with one standard deviation from the average value.

To test cytotoxicity of ADUTDAS, ADUBS, AAZ and SLC-0111 under intermittent hypoxia (HN and HNH); normoxia (N, NN and NNN) and hypoxia (H, HH and HHH), cells were cultured in eight 96-well plates designated as N, H, NN, HN, HH, NNN, HNH and HHH at a density of 5000 cells/well and were allowed to attach and to grow in normal conditions (37 °C, 5% CO_2_ in air) for 24 h. After 24 h of growth in normal conditions, the H, HN, HH, HNH, HHH designated plates were placed in a hypoxic chamber, which was purged and filled with a hypoxic gas mixture (1% O_2_, 5% CO_2_ and 94% N_2_) to induce hypoxia, and the closed chamber with hypoxic plates was kept in the incubator at 37 °C for 48 h. To maintain hypoxic conditions, the chamber was repurged with hypoxia gas mixture every 24 h until the end of the experiment. After another 48 h (day 3), N and H designated plates retrieved and treated with 100 µM of CAIs pre-dissolved in media. Media was changed from remaining plates. Subsequently, H (treated), HH and HHH designated plates were returned to hypoxic chamber, purged with the hypoxia gas mixture, whereas N (treated), NN, NNN, HN and HNH-designated plates were kept under normoxia. After 24 h incubation with treatment, treated N and H plates were retrieved for an MTT assay, as mentioned above. Similarly, at day 5, NN, HN and HH-designated plates were retrieved and treated with the same treatment and incubated in respective growth condition for 24 h and retrieved for an MTT assay. The remaining plates only got a media change before they were returned to their corresponding growth conditions. Lastly, at day 7, NNN, HNH and HHH-designated plates were treated with the same treatment and returned to their respective growth conditions for 24 h. After the incubation period, the plates were retrieved for an MTT assay as mentioned above. Data were reported as the average of four experiments, with one standard deviation from the average value. Statistical comparisons were performed by analysis of variance (ANOVA) using GraphPad Prism 8. *p*-values * *p* < 0.05, ** *p* < 0.01, *** *p* < 0.001 and ns = not significant were considered.

## 4. Conclusions

Using HT-29, MDA-MB-231 and SKOV-3 tumor cell lines grown under normoxic/hypoxic/intermittently hypoxic conditions, we confirmed the carbonic anhydrase IX epitope upregulation in hypoxic conditions and its retention when hypoxic tumor cells are re-oxygenated (intermittent hypoxia). We also correlated the CA IX epitope expression dynamics under normoxic/hypoxic/intermittently hypoxic conditions with tumor cell growth, with the drop in extracellular pH and with the tumor cell killing and tumor growth inhibition by ureido-sulfonamide CAIs and classical acetazolamide CAI. The extracellular pH drop correlated well with the level of CA IX expression, with the intermittent hypoxic cells showing a similar pH drop to fully hypoxic ones. All cancer cells showed higher sensitivity to CA IX inhibitors under hypoxia as compared to normoxia. The tumor cell sensitivity to CAIs under hypoxia and intermittent hypoxia were similar and higher than in normoxia, and appeared to be correlated with the lipophilicity of the CAI. We hope that these observations will help the future development of CA IX inhibitors for cancer treatment.

## Data Availability

The data presented in this study are available in the article. Additional data are available from the corresponding author upon request.
